# Fusion Partner Facilitates Expression of Cell-Penetrating Peptide L2 in *Pichia pastoris*

**DOI:** 10.3390/antibiotics13121207

**Published:** 2024-12-11

**Authors:** Xuan Li, Na Yang, Yuxin Fang, Ruoyu Mao, Ya Hao, Da Teng, Na Dong, Anshan Shan, Jianhua Wang

**Affiliations:** 1 Gene Engineering Laboratory, Feed Research Institute, Chinese Academy of Agricultural Sciences, 12 Zhongguancun Nandajie St., Haidian District, Beijing 100081, China; 2 Innovative Team of Antimicrobial Peptides and Alternatives to Antibiotics, Feed Research Institute, Chinese Academy of Agricultural Sciences, Beijing 100081, China; 3 Key Laboratory of Feed Biotechnology, Ministry of Agriculture and Rural Affairs, Beijing 100081, China; 4 Laboratory of Molecular Nutrition and Immunity, College of Animal Science and Technology, Northeast Agricultural University, Harbin 150038, China

**Keywords:** fusion expression, small ubiquitin-related modifier (SUMO), small fusion tags, cell-penetrating peptide L2, *Pichia pastoris*

## Abstract

Background: L2 is formed by combining the pheromone of *Streptococcus agalactiae* (*S. agalactiae*) and a cell-penetrating peptide (CPP) with cell-penetrating selectivity. L2 has more significant penetration and better specificity for killing *S. agalactiae*. However, the production of AMPs by chemical synthesis is always a challenge because of the production cost. Methods: This study was devoted to the heterologous expression of the cell-penetrating peptide L2 in *Pichia pastoris* using SUMO and a short acidic fusion tag as fusion partners, and the high-density expression of SUMO-L2 was achieved in a 5 L fermenter. Results: The results showed that SUMO-L2 expression in the 5 L fermenter reached 629 mg/L. The antibacterial activity of recombinant L2 was examined; the minimum inhibitory concentration (MICs) and minimum bactericidal concentration (MBCs) of purified L2 were 4–8 μg/mL and 8–16 μg/mL against *S. agalactiae* after 84 h of lysis with 50% formic acid. Conclusions: The findings suggest that SUMO is a suitable fusion tag to express cell-penetrating peptide L2.

## 1. Introduction

*Streptococcus agalactiae* (*S. agalactiae*) is one of the most important pathogens that causes mastitis in dairy cows, streptococcal disease in fish, and even bacterial meningitis and septicemia in newborns [1,2,3]. The high prevalence of *S. agalactiae* worldwide and the rapid emergence of antibiotic-resistant strains highlight the urgent need for new therapeutic interventions [4].

Antimicrobial peptides (AMPs) represent a crucial element of the innate biological defense system, displaying advantages over other antibacterial drugs in terms of high specificity and low resistance to pathogens, which may open up new avenues for the development of antimicrobial agents [5]. Since *S. agalactiae* can enter and colonize intracellularly, it is crucial to identify AMPs capable of penetrating cells to exert their effects. The ability of AMPs to enter the system intracellularly is influenced by various physicochemical factors, including charge, molecular weight, and others [6]. Cell-penetrating peptides (CPPs) are natural or synthetic peptides with the ability to interact with the cell membrane to enter the cell and/or deliver goods. They display analogous characteristics to those of AMPs [7]. L2, a bifunctional AMP, was engineered by Li et al. 2020 by integrating hydrophobic peptide pheromone from *S. agalactiae* (DILIIVGG) with cationic cell-penetrating peptides (KERKKRRR) [8,9]. L2 has been demonstrated to exhibit good cell penetration and a specific antimicrobial effect against *S. agalactiae*, without affecting the host or probiotics [8]. However, the high cost of chemical synthesis and their failure of direct expression greatly limit the clinical application of AMPs [10]. Fusion expression of AMPs using genetically engineered strains can be a good solution to the obstacles.

As a eukaryote, *Pichia pastoris* is a methylotrophic yeast with the advantage of post-translational processing and modification, allowing the secretion of heterologous proteins into the culture medium, which is beneficial for the isolation and purification of recombinant proteins [11,12]. A variety of exogenous proteins have been successfully expressed in *P. pastoris*, which are involved in various industries such as food and medicine [13]. Nevertheless, AMPs produced by heterologous expression are often susceptible to proteolysis and even toxic to the host strain, limiting their druggability and feasibility as a drug [14]. The fusion expression of AMPs provides a solution to these challenges. The well-known glutathione transferase (GST), thioredoxin (Trx), small ubiquitin-associated modifier (SUMO), and a number of other labels have been widely used [15,16]. SUMOs are chaperone proteins that are structurally related to ubiquitin but functionally distinct and highly soluble [17]. SUMOs significantly improve the stability and solubility of exogenous proteins, and are resistant to proteolysis. Furthermore, they significantly increase the expression of recombinant proteins and promote their correct folding. Chen et al. expressed Plectasin by the chaperone protein fusion system, and the results demonstrated that the SUMO tag significantly increased the expression of the target protein, and was superior to TrxA, Intein and GST fusion tags [18]. AMP BSN-37 was successfully expressed by SUMO tag fusion [19]. The fused FLAG-tag is composed of the acidic sequence DYKDDDDK with a net charge of −3. This feature is crucial as it counteracts the positive charge of AMPs, thereby facilitating the formation of stable fusion proteins and mitigating the potential toxicity that highly positively charged AMPs might exert on host cells [7,20].

In this study, the cell-penetrating peptide L2 was expressed by fusion in *P. pastoris* and the effects of different fusion tags on the expression effect were compared at the same time. These findings provide methods and ideas for the high-yield expression of similar peptides.

## 2. Results

### 2.1. Design and Construction of the Recombinant Expression Plasmids pPIC-SUMO-L2 and pPIC-2FLAG-L2

**Figure 1:**
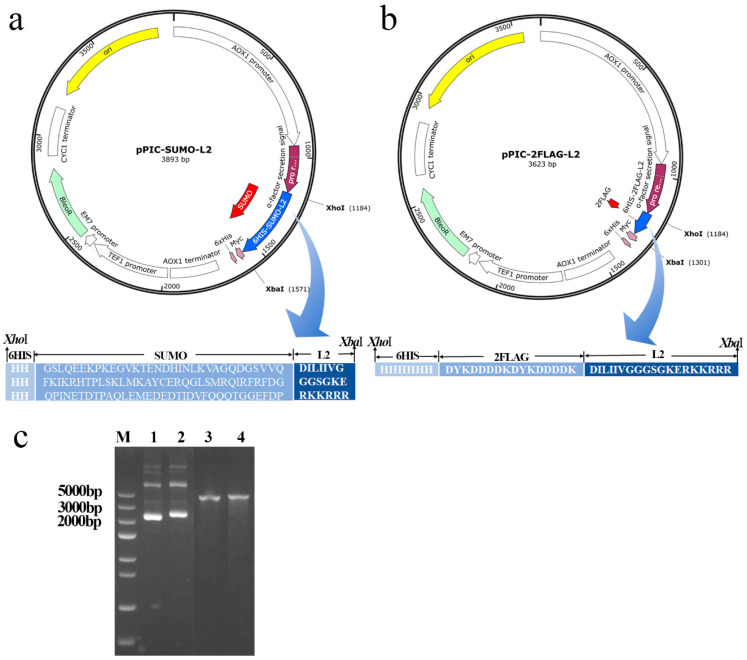
Construction and extraction of recombinant plasmids. (**a**,**b**) Schematic diagram of recombinant expression plasmids pPIC-SUMO-L2 and pPIC-2FLAG-L2. (**c**) Gel analysis of the plasmids pPIC-2FLAG-L2 and pPIC-SUMO-L2. Lane M: Trans5K DNA marker; Lanes 1 and 2: unenzymatically cleaved plasmids pPIC-2FLAG-L2 and pPIC-SUMO-L2; Lanes 3 and 4: linearized plasmids of pPIC-2FLAG-L2 and pPIC-SUMO-L2.

The recombinant expression plasmids pPIC-SUMO-L2 (Figure 1a) and pPIC-2FLAG-L2 (Figure 1b) were extracted from the monoclonal transformed *E. coli* DH5α and linearized by restriction enzyme *Pme*I. The molecular weights of the extracted plasmids were close to 4000 bp (Figure 1c), which is close to the theoretical values of 3893 bp and 3623 bp. The physicochemical property parameters of the fusion protein and L2 are summarized in Table 1.

### 2.2. Methanol-Induced Screening Expression of Recombinant SUMO-L2 and 2FLAG-L2

After the induction of transformants in the microtiter plate, the fermentation broth was harvested and subjected to centrifugation to obtain the supernatant. The protein expression of each transformant was verified by tricine-sodium dodecyl sulfate polyacrylamide gel electrophoresis (Tricine-SDS-PAGE) (Figure S1). The target protein bands of SUMO-L2 were observed to be approximately 15 kDa, which was consistent with the theoretical value (14,123.84 Da). Among the transformants, those numbered 25, 44 and 52 of SUMO-L2 exhibited the darkest protein bands (Figure S1a). The molecular weights of the target bands of 2FLAG-L2 also matched the theoretical values (4951.33 Da), with the most pronounced bands for number 50, 51, and 52 (Figure S1b). Therefore, these six transformants were selected for induced expression at the shake flask level.

**Figure 2:**
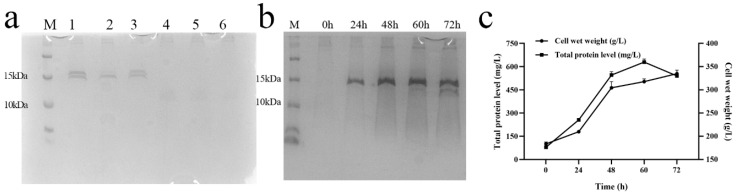
Large-volume induction of recombinant proteins; (**a**) Tricine SDS-PAGE to detect the fermentation supernatant after 120 h of horizontal induction in shaking flasks. Lane M: protein marker (6 μL); Lanes 1–3 represent the transformants of SUMO-L2 named 25, 44, 52 (10 μL), and Lanes 4–6 represent the transformants of 2FLAG-L2 named 50, 51, 52 (10 μL). (**b**) Tricine SDS-PAGE to detect the fermentation supernatant after 72 h of horizontal induction in a 5 L fermenter of SUMO-L2. (**c**) Changes in total protein concentration and wet weight of *P. pastoris* with time during high-density fermentation of SUMO-L2.

The expression of the selected transformants was followed by the determination of protein concentration and analysis of SDS-PAGE. The results (Table 2) showed that the transformants numbered 25 and 52 had a higher protein concentration, but the Tricine-SDS-PAGE results exhibited that the percentage of non-target protein was extremely high. On the other hand, the transformant numbered 44 had a very low percentage of non-target protein (Figure 2a), so the transformant numbered 44 was chosen for the follow-up study.

The final selected transformant was expressed in a 5 L fermenter by high-density fermentation. The SUMO-L2 target band was distinctly visualized on a Tricine-SDS-PAGE gel (Figure 2b). It was observed that the maximum levels of total protein secretion and total biomass were achieved at 629 mg/L and 313 g/L, respectively, following a 60 h induction period (Figure 2c). These values represent a seven-fold increase compared to the protein expression levels in shake flask cultures. Subsequently, the expression level decreased over time.

### 2.3. Purification and Cleavage of SUMO-L2 Fusion Proteins

**Figure 3:**
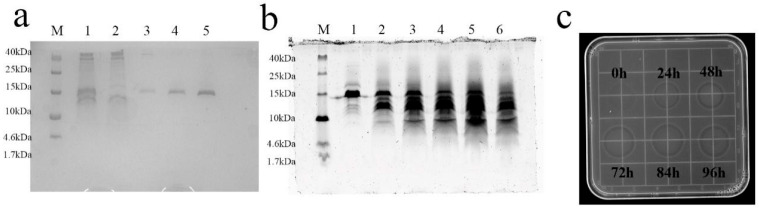
SUMO-L2 purification results by Ni^2+^-chelate affinity column and schematic diagram of formic acid cutting time mapping. (**a**) Tricine-SDS-PAGE results of purified SUMO-L2. Lane M: protein marker; Lane 1: fermentation of unpurified samples; Lane 2: penetration peaks; Lanes 3–4: decontamination peaks; Lane 5: destination peaks; Lane 6: washed peaks. (**b**) Tricine-SDS-PAGE results showed that SUMO-L2 was cleaved using formic acid. Lane M: protein marker; and Lanes 1–6: represent the cleavage time of 0 h, 24 h, 48 h, 72 h, 84 h, and 96 h, respectively. (**c**) Activity test by inhibition zone after formic acid cleavage of SUMO-L2.

The rSUMO-L2 protein was purified using a metal (Ni^2+^)-chelate affinity column. Analysis by Tricine-SDS-PAGE showed a single target band after washing with elution buffer. It is consistent with the location of the target protein (Figure 3a). The mapping of formic acid cleavage conditions to the fusion protein was then performed. The results of the inhibition zone were observed when formic acid was incubated with the fusion protein for 84 h, which also proved that the cutting effect was the best (Figure 3c). Meanwhile, the Tricine-SDS-PAGE results also showed that the target bands were clearest after 84 h (Figure 3b).

### 2.4. Purification and Identification of the Activity of L2

**Figure 4:**
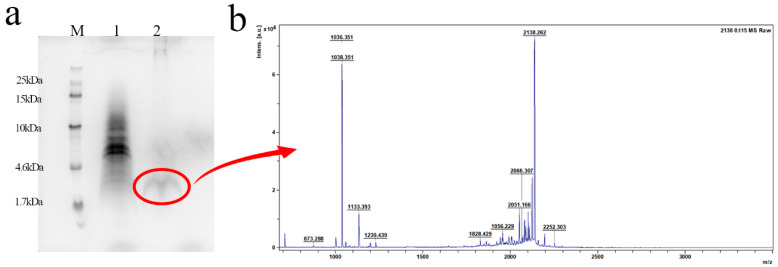
Purification and identification of L2. (**a**) Tricine-SDS-PAGE analysis and purification recovery of L2; bands are in the red circle. M: Spectra™ Multicolor Low Range Protein Ladder. Lane 1: SUMO-L2 after cutting by formic acid; Lane 2: purified product. (**b**) MALDI-TOF MS analysis of purified L2.

The protein bands in the blue box of Figure S2 were recovered using the PAGE Gle Protein Micro Recovery Kit and the obtained products were verified by Tricine-SDS-PAGE, which resulted in the discovery of a clear band at the target molecular weight (Figure 4a). As shown in Figure 4b, it was also verified by MALDI-TOF MS that the molecular weight was 2138.55 Da, which is consistent with the theoretical molecular weight (2138.2 Da). The antimicrobial activity of recombinant L2 was evaluated, the minimum inhibitory concentrations (MICs) of L2 against *S. agalactiae* were 4–8 μg/mL and the and minimum bactericidal concentrations (MBCs) of L2 against *S. agalactiae* were 8–16 μg/mL (Table 3).

## 3. Discussion

*S. agalactiae*, as one of the main pathogens causing mastitis in dairy cows, septicemia in newborns, etc., has a great impact on human and animal health. According to previous research data, about 50–60% of bovine mastitis caused by *S. agalactiae* in Germany and Brazil [21]. So far, antibiotics have been used as an important choice for the treatment of streptococcal infections. In particular, β-lactam antibiotics, macrolides and fluoroquinolones such as ampicillin, penicillin and norfloxacin are the most effective and widely used for the primary clinical treatment [22,23]. However, the bacterial resistance and high residue caused by antibiotics are important factors limiting their use. The lack of target specificity of most antibiotics, which can kill host commensals except the causative organism, also increases the risk of developing resistance for microorganisms [24]. The China Antimicrobial Surveillance Network (CHINET) has revealed that clinical strains of *S. agalactiae* collected during the first half of 2022 exhibited resistance rates of up to 59.7% and 74.5% to clindamycin and erythromycin, respectively [25]. Therefore, extensive research is urgently needed to find a new treatment strategy.

L2 is a cell-penetrating peptide endowed with selective bactericidal properties, demonstrating stability, biocompatibility in vitro, and significant therapeutic efficacy in vivo. The design of L2 was merged with a pheromone from *S. agalactiae* bacteria and a cell-penetrating peptide, known as a transporter carrier. Among them, pheromones are present in almost all *Streptococcus* species and are not only necessary for the exertion of targeting ability, but also an important part of the relationship between streptococcal cell-to-cell intercommunication, even affecting pathogenicity [8,26]. In addition, L2 was found to have multiple bactericidal mechanisms and intracellular antibacterial activity [8]. Therefore, it is anticipated that L2 will not be susceptible to drug resistance. These findings indicate the potential for its application in the treatment of *S. agalactiae* infections and suggest that it is a highly promising candidate.

In the previous report, L2 was obtained by chemical synthesis, which is currently one of the main measures of obtaining AMPs [8]. Nevertheless, the high cost of synthesis AMPs represents a significant obstacle to their clinical application [27]. In contrast, the technique of heterologous expression of proteins can not only solve this difficulty to a certain extent, but also be purposeful, efficient and suitable for large-scale production [28,29]. While a perfect host for a particular protein has yet to be identified, AMPs are still predominantly expressed in bacteria and yeast. The yeast expression system, due to its post-translational modifications, has the unique ability to express AMPs that can form natural conformations, such as disulfide bonds, which help to maintain their potential antimicrobial activity. Human α-defensin 5 [30], cell-penetrating antifungal PAF102 [31], Plectasin [32], etc., have been successfully and efficiently expressed in yeast expression systems. The industrial-scale platforms that can be found in our laboratory for the fermentation and purification of AMPs, with a capacity of 20 and 30 m^3^, respectively, were first established in China in 2019 and 2021 [33]. Furthermore, 49 kg of AMP products with a purity exceeding 92% was harvested. This will undoubtedly facilitate the evaluation of and clinical trials on AMPs that will be conducted in subsequent studies during the drug development process. Given the foundation of our laboratory, the *P. pastoris* expression system became the first choice for L2 expression.

**Figure 5:**
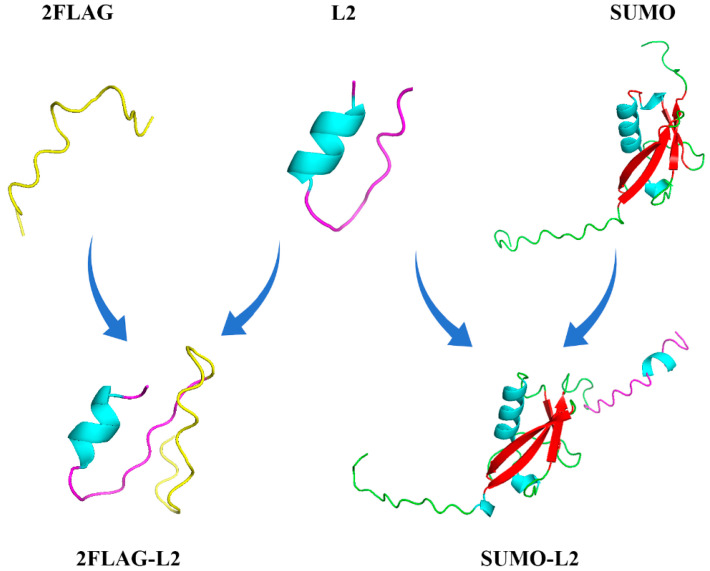
Modeling the secondary structure of L2 and fusion tags using I-TASSER (https://zhanggroup.org/I-TASSER/, accessed on 9 April 2024).

However, not all AMPs are suitable for direct expression in *P. pastoris.* Some AMPs exhibit strong cationic characteristics and are prone to toxicity in the host during expression [12,34]. Currently, the fusion chaperone is one of the most reliable methods to increase the expression of desired proteins [35]. The function of the fusion chaperone is analogous to that of a precursor peptide [36]. In addition to safeguarding the host bacterium and the target peptide, fusion chaperones improve peptide solubility, increase purification efficiency and reduce production costs. Commonly used fusion chaperones include TRX, SUMO, GST, and others. In order to select and design more appropriate fusion tags, it is necessary to understand the physicochemical properties of proteins, including pI, net charge and other physicochemical properties. In previous studies, N6, and Hepcidin25 were successfully expressed by fusion with SUMO protein [37,38]. A small acidic fusion tag (2FLAG: (DYKDDDDK)_2_) was used to express AOD, and its potential antimicrobial activity demonstrates that the tag promotes the formation of a natural conformation of the AOD [20]. The novel dimeric antimicrobial peptide LIG was successfully expressed by a small acidic fusion tag in *P. pastoris*, and the expression level of rLIG was 5.9 mg/L [39]. In this study, L2 was expressed by fusion of the molecular chaperone SUMO and 2FLAG. SUMO-L2 reached the yield of 629 mg/L under 60 h induction in a 5 L fermenter. However, the expression of 2FLAG-L2 was lower than that of SUMO-L2 at the shake flask level (Table 2). SUMO has a typical tertiary structure [40], and the connection of L2 at its C-terminus has no significant effect on its conformation (Figure 5). Nevertheless, the acidic fusion tag FLAG has an unstable random structure, and its instability index changes greatly after its C-terminal attachment to L2 (Table 1), which is indicative of alterations in the spatial structure, leading to a reduction in L2 expression level. A similar result was found with the expression of PR-39-DP with the fusion carrier system calmodulin and xylanase [41]. Consequently, SUMO was deemed a superior candidate for the expression of L2.

SUMO-L2 is cleaved by formic acid. Formic acid cleavage is efficient, gentle, and affordable and does not result in new contaminants compared to enzymatic cleavage [42]. The antimicrobial activity of recombinant L2 (MIC = 4–8 μg/mL) was found to be comparable to that of chemically synthesized L2 [8], despite the latter being C-terminal-amidated, a modification that has been demonstrated to enhance the activity and stability of AMPs.

In summary, SUMO and FLAG tags were used to boost the expression of cell-penetrating peptide L2. The yield of SUMO-L2 was higher than that of 2FLAG-L2 at the shaker level and its total protein concentration reached 629 mg/L in a 5 L fermenter after 60 h of induction. After cleavage by formic acid, L2 exhibited potent antibacterial activity against *S. agalactiae*, comparable to that of chemically synthesized L2, which suggests that SUMO is a suitable fusion tag to express cell-penetrating peptide L2. It has been proven that choosing the right fusion tag is an effective measure to enhance heterologous expression.

## 4. Materials and Methods

### 4.1. Strains, Vectors and Reagents

*Escherichia coli* DH5α (Invitrogen, Beijing, China) was selected as the host strain for vector construction. Plasmid pPICZαA and *P. pastoris* X-33 (preserved in this laboratory) were selected as the cloning vector and expression strain, respectively. The plasmid extraction and DNA purification kits were purchased from Tiangen Biotech Co. Ltd (Beijing, China). PAGE Gle Protein Micro Recovery Kit was purchased from Beijing Solarbio Science & Technology Co. Ltd (Beijing, China). *S. agalactiae* ATCC13813 was purchased from the American Type Culture Collection (ATCC, Manassas, VA, USA). *S. agalactiae* ACCC 61733 was obtained from the South China Sea Fisheries Research Institute of Chinese Academy of Fishery Sciences. *S. agalactiae* CAU-FRI 2, CAU-FRI 3, and CAU-FRI 4, isolated from bovine mastitis, were obtained from China Agricultural University. *S. agalactiae* PBSA0903 isolated from tilapia was obtained from Hainan University. All other reagents were of analytical=grade purity.

### 4.2. Design and Construction of Recombinant Plasmid

The gene sequences of SUMO-L2 and 2FLAG-L2 were optimized based on codon preferences of *P. pastoris*. As shown in Figure 1a,b, *Xho*I and *Xba*I cleavage sites were added on both sides of the target gene sequences, a Kex2 signal peptide recognition cleavage site (Glu-Lys-Arg) was added at the 5′ end, and the termination codon sequences TAATAG were added downstream of the gene sequence. A 6-His-tag was added at the N-terminal of the SUMO-L2 and 2FLAG-L2 protein to facilitate the purification of the recombinant peptide. A formic acid cleavage site was designed between the coding sequences of SUMO and L2 (Figure 1a), and an enterokinase cleavage site was designed between the coding sequences of 2FLAG and L2 (Figure 1b) [31,36]. The optimized gene sequences of SUMO-L2 and 2FLAG-L2 were ligated into pPICZαA following digestion with *Xho*I and *Xba*I, and the recombinant expression vectors of pPIC- SUMO-L2 and pPIC-2FLAG-L2 were constructed, and then transferred into *E. coli* DH5α.

### 4.3. Transformation of P. pastoris X-33 and Screening of Positive Transformants

Recombinant plasmids were extracted using the Plasmid Extraction Kit from Tiangen Biotech Co. Ltd (Beijing, China), and the extracted recombinant plasmids pPIC-SUMO-L2 and pPIC-2FLAG-L2 were linearized using *Pme*I enzyme and detected by agarose gel electrophoresis. After transformation of the linearized plasmid into *P. pastoris* X-33 by electroporation, positive transformants were screened on YPDS plates containing 100 μg/mL zeocin [43].

### 4.4. Expression of SUMO-L2 and 2FLAG-L2 in P. pastoris in 48-Well Plates, Shake Flasks and A 5 L Fermenter Level

The transformants were selected to express the recombinant SUMO-L2 and 2FLAG-L2 in 48-well plates with a final concentration of 0.5% methanol as an inducer. The fermentation broth was collected and centrifuged to obtain the supernatant after induction for 96 h, then Tricine-SDS-PAGE was performed to verify the expression of the peptide [44]. Positive transformants with higher expression were selected for further expansion of the culture in shake flasks. The Tricine-SDS-PAGE and Bradford protein assay were performed to validate and measure the protein concentration.

The positive transformant observed after the second screening was expressed at a high density within the 5 L fermenter, according to the previously established technical method [31]. During the methanol-induced phase, samples were taken every 24 h, and cell pellets and supernatants were collected by centrifugation for cell wet weight and protein analysis.

### 4.5. Purification and Cleavage of the SUMO-L2

The fermentation supernatant was purified by HisTrap TMHP Nickel Column. The Ni^2+^ column was equilibrated with a binding buffer (50 mmol L^−1^ NaH_2_PO_4_, 500 mmol L^−1^ NaCl, 10 mmol L^−1^ imidazole, pH 7.4) and then sampled. After the injection, a gradient elution with elution buffer (50 mmol L^−1^ NaH_2_PO_4_, 500 mmol L^−1^ NaCl, 500 mmol L^−1^ imidazole, pH 7.4) was performed [20]. The elution was monitored by UV280 nm and the penetration and elution peaks were collected sequentially. Analysis was performed by Tricine-SDS-PAGE. Purified fusion proteins were desalted and lyophilized for subsequent experiments. The lyophilised powder of the fusion protein was dissolved in ddH_2_O at a concentration of 2 mg/mL. Formic acid was added to the protein solution at a final concentration of 50%, and the fusion protein was cut at 50 °C for 24 h, 48 h, 72 h, 84 h, and 96 h [36]. The cleavage effect of fusion protein SUMO-L2 was analyzed by Tricine-SDS-PAGE and the antimicrobial activity was validated by the inhibition zone assay to explore the optimal cutting time of the fusion proteins.

### 4.6. Purification and Identification of L2

Purification and recovery of cleaved L2 were performed according to the method of the PAGE Gle Protein Micro Recovery Kit [45]. After an 84 h incubation with formic acid, SUMO-L2 was harvested and processed for Tricine-SDS-PAGE analysis. The gel that contained the target bands (Figure S2) were cut off and decolored until they were nearly colorless, and then ground into fine fragments. The latter steps were performed according to the kit instructions, and purified product was obtained. The molecular weight of the purified product was identified using Tricine-SDS-PAGE and matrix-assisted laser desorption/ionization–time of flight mass spectrometry (MALDI-TOF/TOF (Ultraflextreme, Brucker, Germany)) and analyzed by FlexAnalysis v.3.4 software.

### 4.7. Antimicrobial Activity of L2

MIC and MBC of L2 against *S. agalactiae* were determined according to the Clinical and Laboratory Standards Institute (CLSI) [46]. MIC plates were prepared by culturing the strains in TSB medium until the logarithmic growth stage, diluted in fresh medium to 1 × 10^5^ CFU/mL, and then mixed with a gradient dilution of L2 in 96-well plates. The 96-well plates were incubated at 37 °C for 18–24 h. The liquid in the clearing wells coated the solid medium, and was incubated at 37 °C for 24 h. The concentration represented by the plate with no colony growth was taken as the MBC [47]. All treatment groups were run in triplicate, and sterile saline served as a negative control in the same amount (10 μL).

## Figures and Tables

**Table 1:** Physiochemical parameters of AMP and fusion proteins.

Peptide	Number of Amino Acids	Theoretical Molecular Weight (Da)	Theoretical pI	Positively Charged Residues	Negatively Charged Residues	Instability Index	GRAVY
L2	19	2138.55	11.57	7	2	96.65	−0.926
SUMO	98	11,180.48	4.98	13	18	37.78	−0.965
FLAG	8	1012.98	3.97	2	5	−1.86	−3.325
SUMO-L2	123	14,123.86	7.27	20	20	44.15	−1.068
2FLAG-L2	41	4951.33	6.63	11	12	46.01	−2.195

pI, isoelectric point; GRAVY, grand average of hydropathicity.

**Table 2:** Shake flask level expression of positive transformants.

Positive Transformants	Total Protein Levels (μg/mL)
SUMO-L2 25	129
SUMO-L2 44	86
SUMO-L2 52	92
2FLAG-L2 50	81
2FLAG-L2 51	90
2FLAG-L2 52	77

**Table 3:** MICs and MBCs of L2 against *S. agalactiae*.

Strains	MIC (μg/mL)	MBC (μg/mL)
*S. agalactiae* ATCC 13813	4	8
*S. agalactiae* CAU-FRI 2	8	8
*S. agalactiae* CAU-FRI 3	8	8
*S. agalactiae* CAU-FRI 4	8	16
*S. agalactiae* ACCC 61733	8	8
*S. agalactiae* PBSA0903	8	8

## Data Availability

The original contributions presented in this study are included in the article/Supplementary Materials. Further inquiries can be directed to the corresponding author(s).

## Supplementary Materials

The following supporting information can be downloaded at https://www.mdpi.com/article/10.3390/antibiotics13121207/s1, Figure S1: Tricine-SDS-PAGE to detect protein secretion of transformants screened by well plate; (a) Lane M: protein marker (6 μL); Lanes 1–67 represent each transformant of SUMO-L2 (10 μL). (b) Lane M: protein molecular weight marker (6 μL); Lanes 1–72 represent each transformant of 2FLAG-L2 (10 μL). Figure S2: Tricine-SDS-PAGE results showing that the blue rectangular box is the target band to be recovered.
