# Supplementary material for: Fusion Partner Facilitates Expression of Cell-Penetrating Peptide L2 in Pichia pastoris
Source: Antibiotics (Basel). 2024 Dec 11;13(12):1207. doi: 10.3390/antibiotics13121207 (PMC11672777; doi:10.3390/antibiotics13121207)
Supplement: Supplementary file 1 [file antibiotics-13-01207-s001.zip › antibiotics-3334155-supplementary.pdf]

Supplementary information

Fusion partner facilitates expression of cell-penetrating peptide L2 in *Pichia pastoris*

Xuan Li [1,2,3,†], Na Yang [1,2,3,†], Yuxin Fang [4], Ruoyu Mao [1,2,3], Ya Hao [1,2,3], Da Teng [1,2,3,*], Na Dong [4,*], Anshan Shan [4] and Jianhua Wang [1,2,3,*]

[1] Gene Engineering Laboratory, Feed Research Institute, Chinese Academy of Agricultural Sciences, 12 Zhongguancun Nandajie St., Haidian District, Beijing 100081, China

[2] Innovative Team of Antimicrobial Peptides and Alternatives to Antibiotics, Feed Research Institute, Chinese Academy of Agricultural Sciences, Beijing 100081, China

[3] Key Laboratory of Feed Biotechnology, Ministry of Agriculture and Rural Affairs, Beijing 100081, China

[4] Laboratory of Molecular Nutrition and Immunity, College of Animal Science and Technology, Northeast Agricultural University, Harbin, PR China

* Correspondence: tengda@caas.cn (D.T.); ndong@neau.edu.cn (N.D); wangjianhua@caas.cn (J.W.); Tel.: +86-10-82106081 or +86-10-82106079 (J.W.); Fax: +86-10-82106079 (J.W.)

† These authors contributed equally to this work.

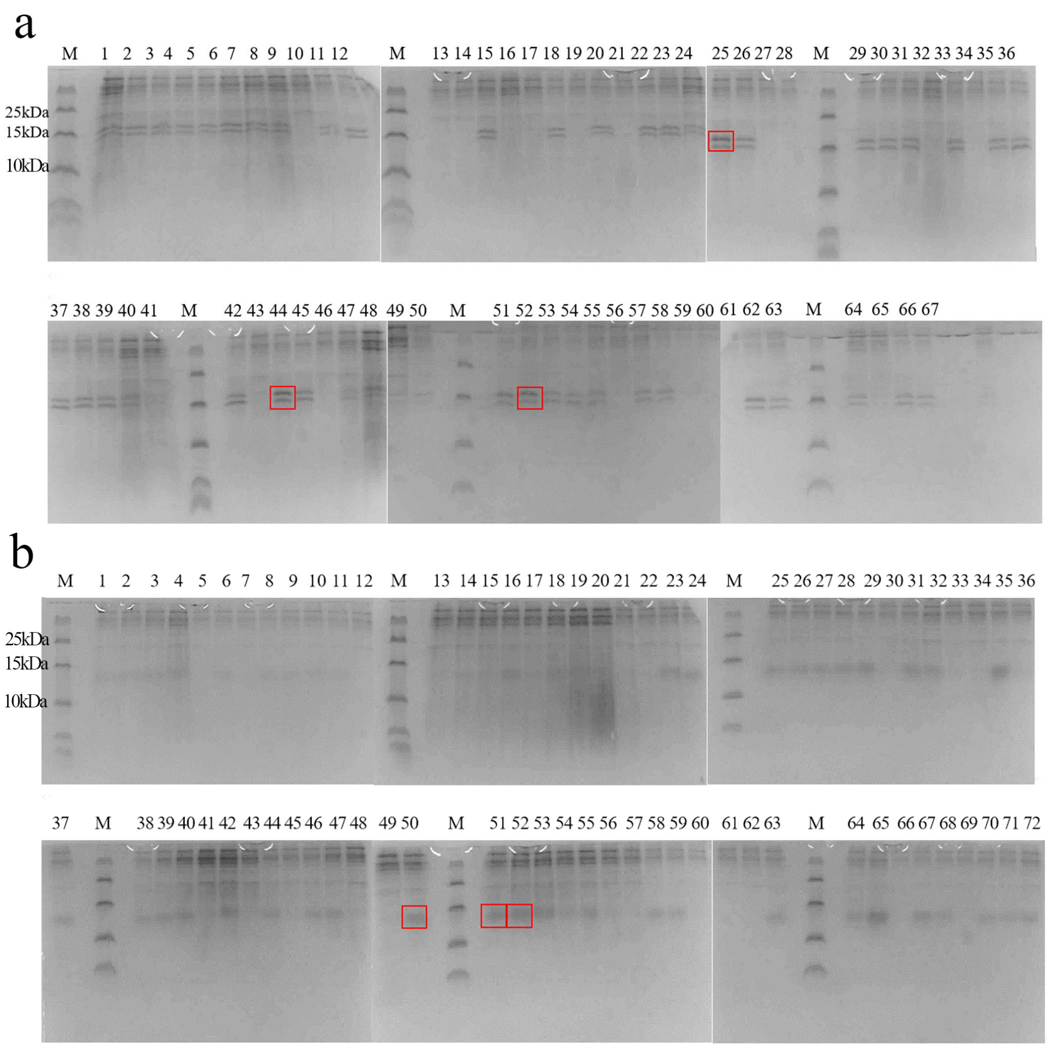

Figure S1. Tricine-SDS-PAGE to detect protein secretion of transformants screened by well plate; (a) Lane M: protein marker (6 μL), Lanes 1-67 represent each transformant of SUMO-L2 respectively (10 μL). (b) Lane M: protein molecular weight marker (6 μL), Lanes 1-72 represent each transformant of 2FLAG-L2 respectively (10 μL).

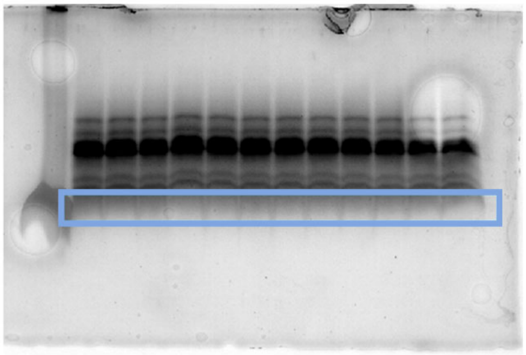

Figure S2. Tricine-SDS-PAGE results show that the blue rectangular box is the target band to be recovered.
